# Adropin: a key player in immune cell homeostasis and regulation of inflammation in several diseases

**DOI:** 10.3389/fimmu.2025.1482308

**Published:** 2025-01-21

**Authors:** Junmin Wang, Ning Ding, Chong Chen, Simin Gu, Jing Liu, Yanping Wang, Liubing Lin, Yiyuan Zheng, Yong Li

**Affiliations:** Shanghai Municipal Hospital of Traditional Chinese Medicine, Shanghai University of Traditional Chinese Medicine, Shanghai, China

**Keywords:** adropin, inflammation, immunity, disease, pathway

## Abstract

Adropin is a secreted peptide encoded by the energy homeostasis-associated gene (ENHO), located chromosome 9p13.3, with a conserved amino acid sequence across humans and mice. Its expression is regulated by various factors, including fat, LXRα, ERα, ROR, and STAT3. Adropin plays a critical role in glucose and lipid metabolism, as well as insulin resistance, by modulating multiple signaling pathways that contribute to the reduction of obesity and the improvement of blood lipid and glucose homeostasis. Additionally, it influences immune cells and inflammation, exerting anti-inflammatory effects across various diseases. While extensive research has summarized the regulation of cellular energy metabolism by adropin, limited studies have explored its role in immune regulation and inflammation. To enhance the understanding of adropin’s immune-modulating and anti-inflammatory mechanisms, this review synthesizes recent findings on its effects in conditions such as atherosclerosis, diabetes, fatty liver, non-alcoholic hepatitis, and inflammation. Furthermore, the review discusses the current research limitations and outlines potential future directions for adropin-related investigations. It is hoped that ongoing research into adropin will contribute significantly to the advancement of medical treatments for various diseases.

## 1 Introduction

Adropin is a secreted peptide encoded by the energy homeostasis-associated gene (ENHO), with expression detected in various tissues, including serum, plasma, liver, kidney, heart, pancreas, small intestine, endothelial cells, and the brain (1–3). This peptide plays a pivotal role in reducing obesity and improving blood lipid and glucose homeostasis by regulating glucose and lipid metabolism, as well as insulin resistance (IR) (4–6). Its mechanism of action involves influencing the insulin metabolic pathway, including activation of the glucose transporter protein (GLUT) receptor and the phosphorylation of protein kinase B (AKT) (7). Additionally, adropin modulates lipid metabolism by regulating the expression of liver disease-related genes and peroxisome proliferator-activated receptor gamma (PPARγ), a key regulator of lipogenesis. Activation of PPARγ can reduce macrophage infiltration and inflammation in adipose tissue (2, 8). As a membrane-bound protein, adropin also regulates intercellular communication through molecular signaling. It activates the PI3K-AKT and ERK1/2 pathways via vascular endothelial growth factor 2 (VEGFR 2), upregulating endothelial nitric oxide synthase (eNOS), enhancing nitric oxide (NO) production, improving endothelial cell function, and decreasing endothelial permeability, which subsequently reduces TNF-α-related apoptosis (9). NO plays an essential immunomodulatory role by inhibiting the adhesion of monocytes and leukocytes to endothelial cells.

Although adropin’s role in regulating carbohydrate and lipid metabolism has been well-established, recent studies highlight its anti-inflammatory effects across multiple tissues (4, 10–12). Adropin influences macrophage polarization by modulating their cellular energy metabolism and protects Treg cells from reactive oxygen species (ROS)-induced apoptosis through its antioxidant properties (8). A deficiency in adropin can disrupt immune cell function and inflammatory pathways, impairing the immune system’s regulatory capacity and promoting inflammation (13). Therefore, adropin preserves immune system homeostasis and exerts anti-inflammatory effects in a variety of conditions, including atherosclerosis (9), diabetes (14–16), non-alcoholic fatty liver disease (NAFLD) (6, 17), non-alcoholic steatohepatitis (NASH) (10), and inflammatory bowel disease (18). As a promising target for treating immune and inflammation-related diseases, adropin holds significant therapeutic potential. This review summarizes recent advancements in the understanding of adropin’s role in inflammation and immune regulation in related diseases, offering insights to guide future research in this field.

## 2 Structure and function of adropin

Adropin is a newly discovered 76-amino acid polypeptide identified by Kumar et al. (2). The first 1-33 amino acids constitute a secreted signal peptide (2), while the biologically active region spans amino acids 34-76 (7). The N-terminal (amino acids 1-9) is cytoplasmic, the middle region (amino acids 9-30) spans the membrane, and the C-terminal (amino acids 30-76) is extracellular (19, 20). Adropin has a molecular weight of 4.499 kDa, and its encoding gene, ENHO, is linked to energy homeostasis and lipid metabolism, which is why it was named ENHO (2). The ENHO gene is located on chromosome 9p13.3 and consists of two exons and one intron (2). Notably, the amino acid sequence of adropin is 100% conserved across human, mouse, and rat species (21).

Extensive research has explored the functional role of adropin, particularly in mechanisms related to increased obesity, IR, and glucose and lipid metabolism (5, 22, 23). Studies indicate that adropin promotes glucose metabolism by enhancing glucose utilization in mice, a process involving the regulation of the insulin pathway (7). Furthermore, adropin is involved in lipid metabolism, including the reduction of serum total cholesterol (TC), triglycerides (TG), and low-density lipoprotein cholesterol (LDLC) levels (4). As a membrane-bound protein, adropin also regulates intercellular molecular communication and participates in disease development. Additionally, adropin has been found to influence immunity and inflammation, exerting anti-inflammatory effects across various tissues.

## 3 Regulation of adropin expression

Adropin is secreted and bound to cell membranes, where its expression is regulated by factors such as fat, liver X receptor alpha (LXRα), estrogen receptor alpha (ERα), and regulator of reprogramming (ROR). Kumar et al. discovered that adropin expression was significantly elevated in C57BL/6J mice on a high-fat diet (HFD) compared to controls. Conversely, fasting reduced adropin expression in these mice (2). Additionally, mice fed a high-fat, low-carbohydrate diet exhibited elevated adropin levels, while those on a low-fat and high-carbohydrate diet showed reduced adropin levels (24). These observations suggest that adropin expression is closely tied to dietary fat intake. LXR, a nuclear receptor, serves as both and a blood lipid and blood glucose sensor (25). Treatment with the LXRα agonist (GW3965) in diet-induced obese mice led to a reduction in Enho mRNA expression in the liver, indicating that liver ENHO activity is regulated by LXRα (2, 26). Another study highlighted that estrogen regulates liver adropin, with ovariectomized (OVX) mice treated with estrogen showing increased hepatic Enho expression, driven by estrogen-dependent binding of ERα to Enho (27). Furthermore, research has shown that Enho expression follows a rhythmic pattern in the liver of male mice, peaking during the dark phase when food consumption is at its highest. This expression is associated with the transcriptional activation of the circadian clock genes, RORα/γ (28).

## 4 Signaling pathways regulated by adropin

**Figure 1:**
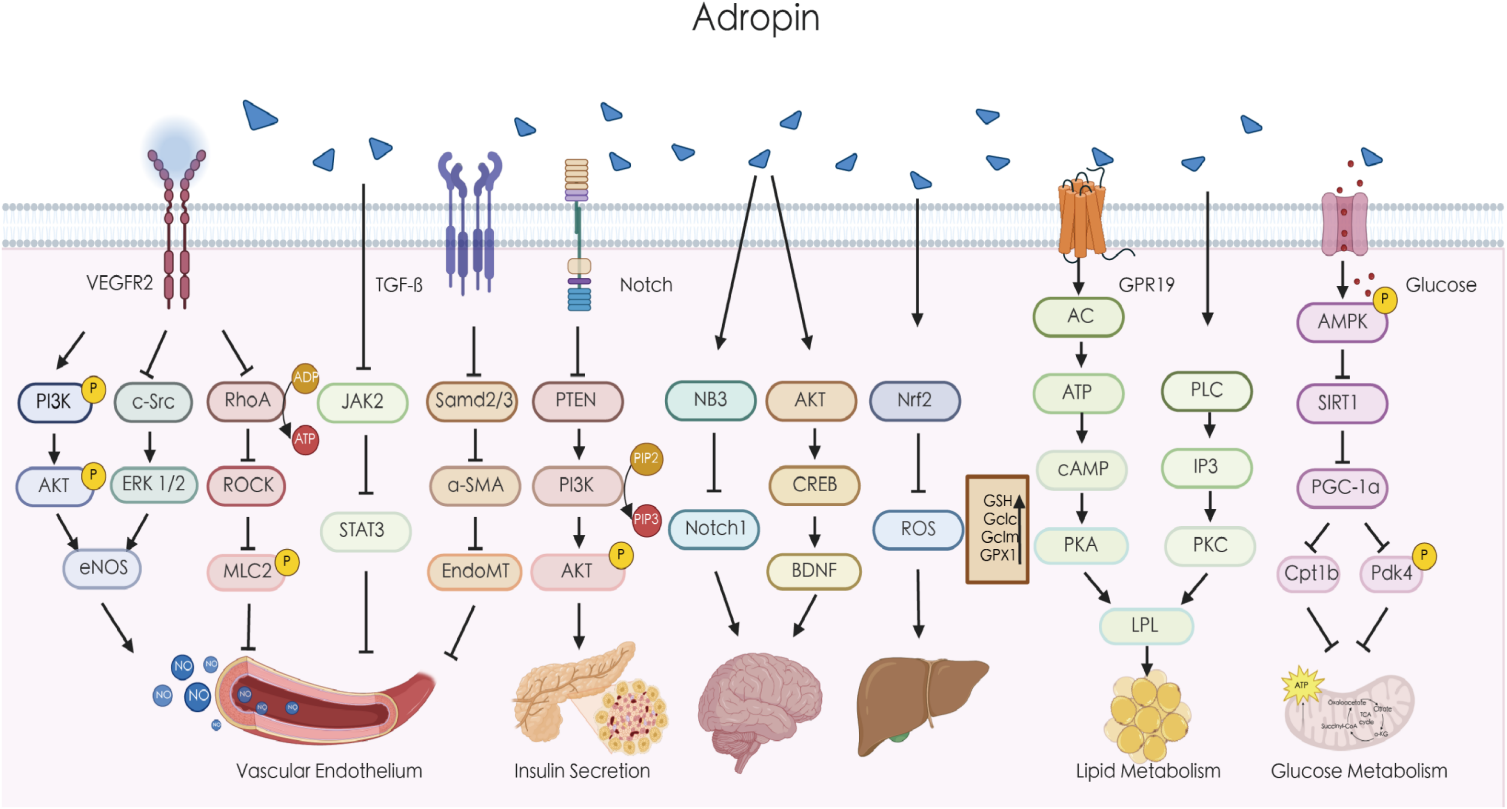
Adropin regulates multiple signaling pathways. Adropin can upregulate the expression of eNOS through the VEGFR2/PI3K/AKT or VEGFR2/c-Src/ERK1/2 pathway, increase the release of NO, and improve endothelial cell function. Adropin can also reduce endothelial cell permeability by inhibiting the ROCK/MLC2 signaling pathway. Adropin can inhibit endothelial calcification by suppressing the JAK/STAT3 signaling pathway. Adropin may alleviate atherosclerosis by inhibiting EndoMT through the TGF-β/Smad2/3 signaling pathway. Adropin downregulates PTEN through the Notch signaling pathway and may increase the basal level of PI3K to increase insulin-induced AKT phosphorylation. Adropin can regulate the function of brain cells by modulating the NB3/Notch1 and AKT/CREB/BDNF pathways. Adropin can improve liver function by activating the Nrf2 pathway to reduce oxidative stress. Adropin also improves fat metabolism by regulating LPL via the cAMP/PKA and PLC/IP3/PKC pathways. In addition, Adropin reduces the expression of PGC-1α by inhibiting SIRT1, thereby downregulating Cpt1b and Pdk4 to regulate glucose oxidation. AKT, protein kinase B; α-SMA, alpha-smooth muscle actin; AMPK, AMP-activated protein kinase; BDNF, brain-derived neurotrophic factor; cAMP, cyclic adenosine monophosphate; CREB, cyclic AMP response element-binding protein; eNOS, endothelial nitric oxide synthase; ERK, extracellular signal-regulated kinase; EndoMT, endothelial-mesenchymal transition; GPR19, G-protein coupled receptor 19; IP3, inositol trisphosphate;JAK, Janus kinase; LPL, lipoprotein lipase; MLC2, myosin light chain kinase; NB3, contactin 6;Nrf2,nuclear factor erythroid 2-related factor 2; PI3K, phosphatidylinositol-3 kinase; PDH, pyruvate dehydrogenase; PDK4, pyruvate dehydrogenase kinase 4; PKC, protein kinase C; PLC, phospholipase C; PTEN, phosphatase and tensin homolog; PKA, protein kinase A; PGC-1α,peroxisome proliferator-activated receptor-gamma coactivator-1alpha; RhoA, ras homolog gene family member A; SIRT1, sirtuin 1; STAT3,signal transducer and activator of transcription 3; TGF-β, transforming growth factor beta.

Adropin, a membrane-bound protein, plays a significant role in regulating intercellular communication (**Figure 1**). In the context of glycolipid metabolism, studies have demonstrated that adropin downregulates peroxisome proliferator-activated receptor gamma coactivator-1 (PGC-1α) expression by inhibiting sirtuin 1, leading to the suppression of carnitine-palmitoyl transferase 1b (CPT 1b) and pyruvate dehydrogenase kinase 4 (PDK4). This cascade effectively gatekeeps fatty acid oxidation and glucose oxidation (29–31). In cardiomyocytes, adropin activates G-protein coupled receptor 19 (GPR19), triggering MAPK-mediated phosphorylation, which in turn downregulates the phosphorylation of PDK4 and pyruvate dehydrogenase (PDH) (32). Gao et al. showed that adropin reduces phosphatase and tensin homolog (PTEN) expression through the Notch signaling pathway in muscle tissue, potentially elevating the basal level of PIP3 (phosphatidylinositol 3,4,5-trisphosphate) and enhancing insulin-induced Akt phosphorylation (7). On the other hand, Chen et al. observed that adropin treatment in HFD mice activates the AMPK pathway by inhibiting PP2A, thereby reducing hepatic glucose production in the context of IR (22). Furthermore, adropin stimulates lipoprotein lipase (LPL) gene expression in tilapia liver cells through the activation of cAMP/PKA and PLC/IP3/PKC cascades (33). In NASH mice, adropin was found to activate the Nrf2 signaling pathway, reducing ROS production in hepatocyte mitochondria and thereby protecting against liver damage (10).

In addition to its role in metabolism, adropin also influences vascular function. Research indicates that adropin reduces endothelial cell permeability by inhibiting the ROCK-MLC2 signaling pathway (34). Sato et al. found that adropin inhibits the proliferation of vascular smooth muscle cells (VSMCs) by downregulating the c-Src/ERK1/2 pathway, while simultaneously upregulating the PI3K-AKT pathway to enhance the expression of fibronectin and elastin, thus stabilizing atherosclerotic plaques and promoting vascular elasticity (8). Furthermore, adropin inhibits the osteogenic differentiation of vascular smooth muscle cells VSMCs and reduces vascular calcification by activating the JAK2/STAT3 signaling pathway (35). In ApoE^–/–^/Enho^–/–^ mice, adropin administration mitigated atherosclerosis, likely through the suppression of endothelial-to-mesenchymal transition (EndMT) via TGF-β/Smad2/3 signaling cascade (36). Adropin’s effects extend to the central nervous system, where it functions as a membrane-bound protein that influences body activity and movement coordination via the NB3/Notch pathway. It plays a critical role in cerebellar development in mice (20). Additionally, adropin enhances spatial memory in rats by modulating the AKT/CREB/BDNF signaling pathway (37). In diabetic rats, adropin treatment reduces lung damage by inhibiting the RhoA/ROCK pathway, thereby alleviating apoptosis, inflammation, oxidative stress, and lung tissue fibrosis (38). These observations collectively highlight adropin’s broad spectrum of molecular activities and its essential role in both physiological and pathological processes throughout the body.

## 5 Adropin regulates immunity and inflammation

### 5.1 Adropin regulates immune cells

**Figure 2:**
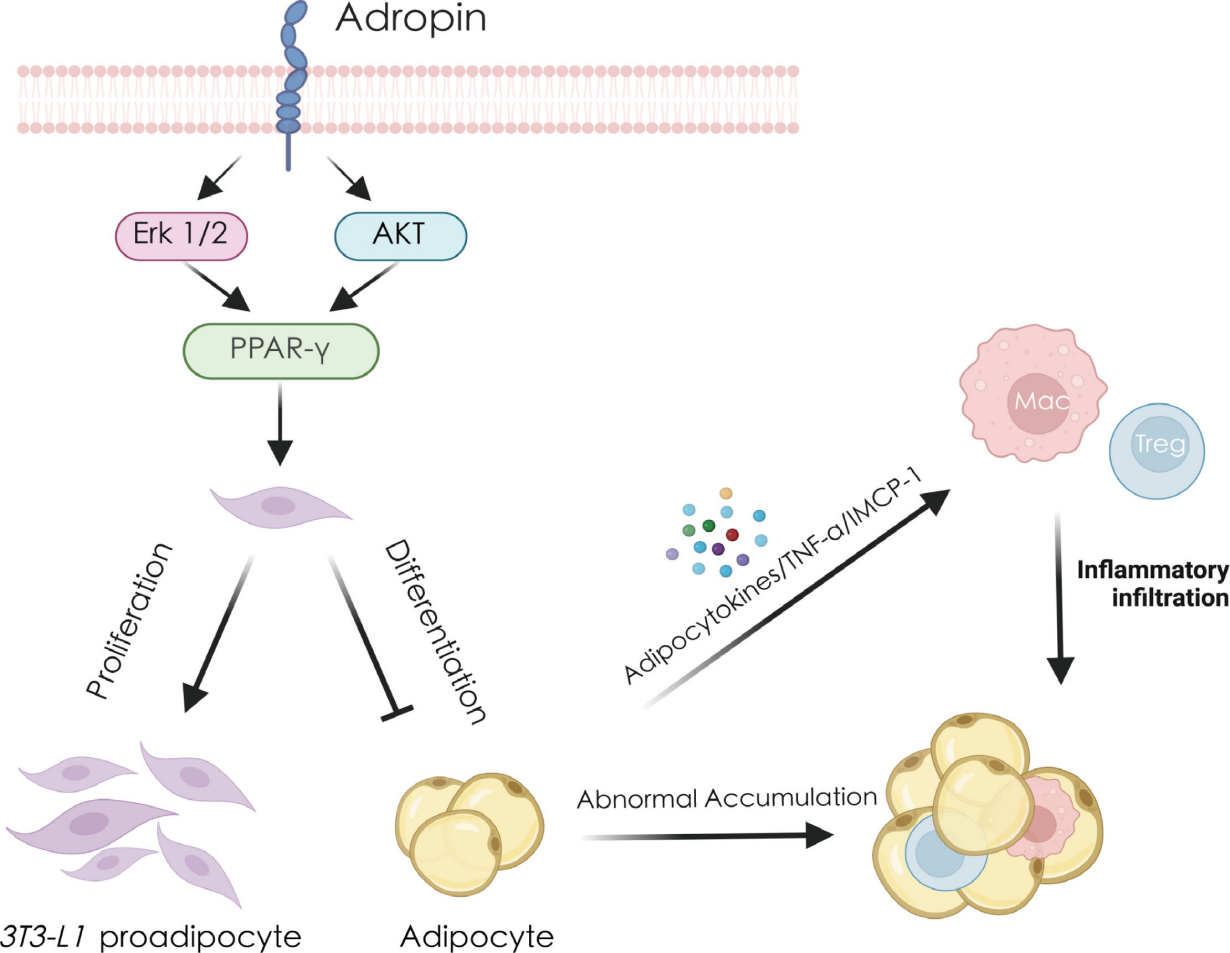
Adropin regulates adipocytes and thereby reduces inflammation and immune response. Adropin promotes the expression of PPAR-γ by activating the ERK1/2 and AKT pathways, stimulating the proliferation of 3T3-L1 preadipocytes and inhibiting their differentiation into mature adipocytes. Excessive accumulation of mature adipocytes will secrete large amounts of TNF-α, MCP-1 and other cytokines, recruit macrophages, Tregs cells, cause immune cell infiltration and ultimately cause fat inflammation. AKT, protein kinase B; ERK1/2, extracellular regulated kinase 1/2; MCP-1, monocyte chemoattractant protein-1; PPAR-γ, peroxisome proliferator-activated receptor gamma; TNF-α, tumor necrosis factor alpha.

Macrophages, key components of the innate immune system, play an essential role in maintaining tissue homeostasis. Upon encountering various stimuli, macrophages become activated and polarized into distinct phenotypes with specific functions (39). M1 macrophages promote and sustain inflammation by secreting pro-inflammatory cytokines, while M2 macrophages have anti-inflammatory properties and aid in tissue repair (40, 41). The metabolism of these cells, particularly lipid metabolism, has a profound influence on their function (42). Studies have shown that the visceral adipose tissue is associated with macrophages in an inflammatory state, where pro-inflammatory macrophages infiltrate adipose tissue, contributing to inflammation and IR (43–45). Macrophages are key drivers of increased expression of inflammatory cytokines in adipose tissue (42), and the number of macrophages in this tissue positively correlates with fat content. The removal of adipose tissue can reduce whole-body inflammation (46, 47). The nuclear receptor PPARγ is more closely associated with lipogenesis and lipid storage, in contrast to PPAR-α and PPAR-β/δ, which primarily regulate fatty acid oxidation (48, 49). Recent research has shown that adropin can promote the repolarization of macrophages from the M1 phenotype to the M2 phenotype, improving the lipid metabolism in macrophages. This process is mediated by the activation of PPARγ (8). In endothelial cells, adropin reduces the inflammatory response of monocyte-derived macrophages by upregulating PPAR-γ expression (50). Dodd et al. further demonstrated that adropin reduces endothelial cell monolayer permeability and diminishes MCP-1-induced macrophage migration following exposure to cell-free hemoglobin (51). Additionally, adropin inhibits the differentiation of 3T3-L1 preadipocytes into mature adipocytes through the ERK1/2 and AKT pathways, reducing fat accumulation and macrophage infiltration, ultimately mitigating inflammation (**Figure 2**) (50). These findings suggest that adropin modulates macrophage polarization and function by influencing cellular energy metabolism pathways, particularly lipid metabolism.

Treg cells play a pivotal role in regulating the inflammatory state of adipose tissue. In obesity, macrophage infiltration into adipose tissue triggers chronic inflammation. Adipocytes release cytokines such as TNF-α and MCP-1, which recruit both macrophages and Tregs, exacerbating adipose inflammation (52). Tregs are crucial for modulating immune-mediated inflammation. Studies have demonstrated a significant reduction in Treg numbers in adipose tissue, with immune dysregulation contributing to adipose inflammation in obese mice. Thus, Tregs are vital in maintaining metabolic homeostasis (53, 54). Gao et al. reported that adropin gene knockout in C57BL/6J mice resulted in reduced phosphorylation of eNOS (Ser1177) and Akt1 (Ser473) and a loss of Treg cells (12). Similarly, Chen et al. found that adropin deficiency aggravated Treg cell depletion and contributed to the development of fatty pancreas (FP) and type 2 diabetes mellitus (T2DM) in mice fed HFD (55). Additionally, studies indicate that elevated oxidative stress in the fatty liver induces Treg cell apoptosis, diminishing the liver’s Treg population and impairing the suppression of inflammatory responses (17, 56). Chen et al. demonstrated that adropin activates Nrf2 signaling in NASH, reducing ROS production in liver mitochondria. This mechanism likely protects mitochondrial function, mitigates oxidative stress and apoptosis, and thereby prevents liver damage and the progression of NASH (10). These observations suggest that adropin can mitigate ROS-induced Treg cell apoptosis by counteracting oxidative stress.

However, further research is needed to explore the regulatory effects of adropin on other immune cell types through distinct signaling pathways.

### 5.2 Adropin regulates inflammation

In addition to modulating inflammation through immune regulation, adropin also influences the expression of inflammatory factors, although the precise molecular mechanisms remain unclear. Chen et al. observed that in the NASH mouse model, adropin nuclear gene knockout led to increased expression of inflammatory markers, including F4/80, CD45, MCP-1, TNF-α, and IL-6, compared to wild-type mice (10). Furthermore, adropin treatment promoted endothelial cell proliferation, migration, capillary-like tube formation, reduced permeability, and mitigated TNFα-induced apoptosis (9). In addition, adropin treatment reduced the expression of pro-inflammatory cytokines IL-1β, IL-6, and TNF-α in methionine-choline-deficiency (MCD) diet-induced NASH mice (10). Other studies have demonstrated that adropin significantly decreased the mRNA expression of TNF-α, IL-6, and inducible NOS in the pancreatic tissue of diabetic rats (15). Collectively, these findings suggest that adropin possesses anti-inflammatory potential.

## 6 Adropin regulates inflammation and immune-related diseases

**Figure 3:**
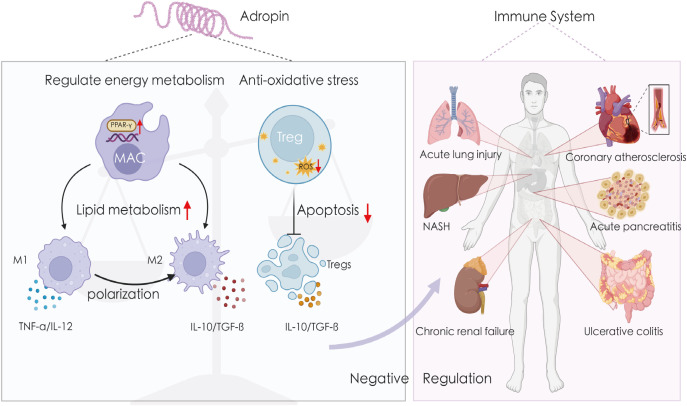
Adropin exerts anti-inflammatory effects in a variety of tissues. Adropin affects macrophage polarization by regulating cellular energy metabolism and inhibits ROS-induced apoptosis of Tregs cells by resisting oxidative stress. Therefore, it can maintain the negative regulation of the immune system and exert anti-inflammatory effects in atherosclerosis, fatty inflammation, non-alcoholic hepatitis, lung injury, inflammatory bowel disease, chronic renal failure, etc. IL-10, interleukin-10; IL-12, interleukin-12; NASH, non-alcoholic hepatitis; TGF-β, transforming growth factor beta; TNF-α, tumor necrosis factor alpha.

Adropin plays a significant role in the development of various metabolic diseases by regulating glucose oxidation, lipid metabolism, and IR. (6, 57). However, studies also indicate that adropin-mediated immune and inflammatory regulation is involved in the pathogenesis of several metabolic and non-metabolic diseases, such as atherosclerosis (36), diabetes (58), NAFLD (59), gastric ulcers (60), and inflammatory bowel disease (18) (**Table 1**, **Figure 3**).

**Table 1:** The specific molecular mechanisms of Adropin regulating different diseases.

Disease	Mechanism	Impact	Ref
Atherosclerosis	Adropin→VEGFR2/PI3K/AKT, VEGFR2/ERK1/2↑→ eNOS↑→ NO↑→Improve endothelial function and promote angiogenesis; Adropin→TNF-α↓→Mononuclear/macrophage inflammatory response↓; Adropin→PPAR-γ↑→Macrophage M1 polarizes toward M2↑→Inflammation↓;	Alleviate	(9, 61, 62)
AP-ALI	Adropin→PPAR-γ↑→Macrophage infiltration↑→Inflammation↓;	Alleviate	(63)
Inflammatory bowel disease	Adropin→PPAR-γ↑→Macrophage infiltration↑→Inflammation↓;	Alleviate	(64)
MPO-ANCA-related lung injury	Adropin↓→eNOS, AKT1, Tregs↓→Inflammation↑;	Aggravate	(12)
Colon cancer	Adropin(low dose)→glucose utilization↑→Anti-tumor↑; Adropin(high dose)→CPT1α↑→Anti-tumor↓;	Alleviate/ Aggravate	(65, 65)
NASH/NAFLD	Adropin→IL-1β, IL-6, TNF-α↓→Inflammation↓; Adropin→Nrf2↑→Gclc, Gclm, Gpx1, GSH↑ → ROS↓ → Inflammation↓;	Alleviate	(10, 10, 66)
Complications of diabetes	Adropin→RhoA/Rock, IL-6, TNF-α, ROS↓→Inflammation↓; Adropin→SEBP-1, ADRP↓→Lipid deposition↓	Alleviate	(38, 67)
Chronic renal failure	Adropin→G-CSF, IFN-γ, IL-4, IL-5, IL-10, IL-12, IL-17A, and GRO-α, TIMP-1, MMP-2/3↓→Inflammation and fibrosis↓;	Alleviate	(68, 69)
Obstructive sleep apnea	patient serum Adropin↓→sVAP-1, IL-6, TNF-α and CRP↑;	Alleviate	(70)
PCOS	patient serum Adropin↓→TNF-α↑;	Alleviate	(71)
Multiple sclerosis	patient serum Adropin↓→unclear	Alleviate	(72, 73)

NASH, non-alcoholic hepatitis; NAFLD, non-alcoholic fatty liver disease; ROS, reactive oxygen species; PPARγ, peroxisome proliferator-activated receptor gamma; VEGFR 2, vascular endothelial growth factor 2; TNF-α, tumour necrosis factor alpha; PI3K, phosphatidylinositol-3 kinase; Nrf2, nuclear factor erythroid 2-related factor 2; eNOS, endothelial nitric oxide synthase; MCP-1, monocyte chemoattractant protein-1; CPT1α, carnitine Palmitoyltransferase 1; CRP, C-reactive protein; CREB, cyclic AMP response element-binding protein; Rock, Rho-associated coiled-coil containing protein kinase; RhoA, ras homolog gene family member A; AP-ALI, acute pancreatitis-associated lung injury; MPO-ANCA, myeloperoxidase antineutrophil cytoplasmic autoantibodies.→, promote; ↑, upregulate; ↓, downregulate.

### 6.1 Atherosclerosis

In the cardiovascular system, atherosclerosis is a chronic inflammatory condition of the arteries. Research has shown that adropin can upregulate eNOS expression through the VEGFR2-PI3K-AKT or VEGFR2-ERK1/2 pathways, facilitating NO release, which improves endothelial cell function and promotes neovascularization (9). NO exerts an immunomodulatory effect by inhibiting the adhesion of monocytes and leukocytes to endothelial cells (61, 62). Moreover, adropin inhibits TNFα-induced THP-1 monocyte adhesion to human umbilical vein endothelial cells (HUVECs) and suppresses the inflammatory response in endothelial cells and monocytes/macrophages by preventing their interaction (9). Notably, in animal models of atherosclerosis, adropin was found to promote macrophage polarization from M1 to M2 by upregulating PPAR-γ, thus reducing monocyte/macrophage infiltration (8).

### 6.2 Acute pancreatitis-associated lung injury

AP-ALI is a severe complication associated with acute pancreatitis. Serum adropin levels are markedly reduced in the patients with AP-ALI. Studies using animal models of AP-ALI have shown that adropin gene knockout (Adro-KO) results in increased macrophage infiltration, fibrosis, and apoptosis in lung tissue. More importantly, adropin modulates the phosphorylation of PPARγ in lung macrophages, thereby promoting M2 polarization and attenuating the severity of AP-ALI (63).

### 6.3 Inflammatory bowel disease

Decreased serum adropin levels have been observed in 55 patients with inflammatory bowel disease compared to healthy controls (18). Adropin deficiency exacerbates the pathological phenotype in TNBS-induced colitis. primarily by disrupting the balance of macrophage phenotypic distribution within the colon and mesenteric tissues. This disruption results in an increased presence of M1 macrophages, contributing to the progression of colitis. Intervention with adropin in macrophages, coupled with RNA-seq and metabolomic analysis, revealed that adropin regulates the macrophage lipid metabolism via PPARγ, thereby promoting the repolarization of macrophages from M1 to M2 (64).

### 6.4 MPO-ANCA-related lung injury

Myeloperoxidase (MPO)-antineutrophil cytoplasmic autoantibodies (ANCA)-associated vasculitis often leads to life-threatening alveolar hemorrhage or fibrosis. In patients with MPO-ANCA-related lung injury, serum adropin levels were significantly lower than those in healthy controls. Investigation of the underlying mechanisms in animal models demonstrated that adropin knockout mice exhibited reduced phosphorylation of eNOS and AKT1, alongside a loss of Treg cells (12).

### 6.5 Colon cancer

Adropin also plays a role in cancer development. A recent study demonstrated that transfection of the ENHO gene into colon cancer (MC38) cells suppressed tumor growth *in vivo* while promoting an increase in M1 macrophages. Treatment with low- -dose adropin in isolated macrophages enhanced mitochondrial ROS-mediated inflammasome activation (65). Notably, while low doses of adropin stimulated macrophage antitumor activity, high doses had the opposite effect. This differential response may be attributed to low-dose adropin promoting glucose utilization, while high-dose adropin upregulates CPT1α expression in macrophages. Thus, varying concentrations of adropin within macrophages in cancer cells or tumor tissues may modulate CRC progression through distinct mechanisms (65).

### 6.6 NASH/NAFLD

Adropin treatment has been shown to mitigate liver cell damage in NASH and NAFLD by reducing inflammation and oxidative stress (10). For instance, Chen et al. reported that, in addition to decreasing liver lipid content, adropin treatment reduced the expression of pro-inflammatory cytokines, including IL-1β, IL-6, and TNF-α, in MCD diet-induced NASH mice (10), indicating its anti-inflammatory effects in NASH. Furthermore, adropin knockout (AdrKO) exacerbated hepatic steatosis, inflammation, and fibrosis, while adropin treatment alleviated these conditions by promoting the expression of Gclc, Gclm, and Gpx1, as well as increasing glutathione (GSH) levels in an Nrf2-dependent manner, thereby preventing NASH progression in mice (10). This suggests that adropin also contributes to the reduction of ROS production in liver mitochondria. Excessive ROS production has been shown to drive inflammation (74), and the activation of the NLRP3 inflammasome through ROS plays a key role in the progression of NASH (75, 76). Yang et al. demonstrated that exercise significantly reduced the expression of NLRP3 inflammasome components in NASH mice, decreased caspase-1 activity, normalized IL-1β production, and inhibited ROS overproduction, a process linked to adropin induction (66). Additionally, elevated oxidative stress levels in fatty liver can induce Treg cell apoptosis and reduce the number of Tregs in the liver, thereby diminishing their anti-inflammatory effects (17, 56). Thus, controlling oxidative stress can also mitigate inflammation. Notably, serum adropin levels were found to be reduced in patients with NAFLD (77), suggesting that adropin may also be involved in the development of NAFLD. Consistent with its action in NASH, adropin overexpression or treatment in NAFLD animal models has been shown to alleviate palmitic acid-induced oxidative stress in hepatocytes (78). Interestingly, Meda et al. observed that under HFD conditions, hepatic adropin induction was negatively correlated with the expression of lipogenic genes and fatty liver in female mice, with this effect being dependent on hepatic ERα (79). More importantly, female-specific induction of adropin under HFD enhances the liver’s response to oxidative stress, helping to counteract ROS production and the inflammatory processes that promote NAFLD progression (79).

### 6.7 Complications of diabetes

In the context of diabetic complications, Rizk et al. demonstrated that adropin treatment can inhibit the RhoA/ROCK pathway, apoptosis, inflammatory responses (IL-6, TNF-α), oxidative stress (malondialdehyde, 8-oxo-20 -deoxyguanosine, reduced glutathione, superoxide dismutase), and lung tissue fibrosis, thereby mitigating diabetic lung injury (38). This positions adropin as promising therapeutic agent for managing diabetes-related injuries. Additionally, Yu et al. found that adropin encapsulated in ROS-responsive nanocapsules improved renal lipotoxicity in diabetic mice, primarily by effectively controlling blood glucose and lipid levels. It also downregulated lipogenic proteins SEBP-1 and ADRP in diabetic nephropathy models, alleviating lipid deposition in renal tissue. Concurrently, adropin inhibited excessive ROS production, protecting mitochondria from damage and improving renal function (67). These observations underscore the critical role of adropin in modulating oxidative stress and its potential impact on the progression of diabetes.

### 6.8 Chronic renal failure

In rats with adenine-induced chronic renal failure, adropin treatment was shown to reduce the expression of several pro-inflammatory cytokines, including G-CSF (granulocyte colony-stimulating factor), IFN-γ, IL-4, IL-5, IL-10, IL-12, IL-17A, and GRO-α (growth-related oncogene-alpha) (68). Another study revealed that adropin treatment in chronic renal failure rat models also decreased renal damage markers such as NGAL (neutrophil gelatinase-associated lipocalin), TIMP-1, IL-17A, IL-33, MMP-2, and MMP-3, while increasing MMP-13 levels (69).

### 6.9 Obstructive sleep apnea

Notably, serum adropin levels were significantly reduced in individuals with obstructive sleep apnea, accompanied by elevated levels of soluble vascular adhesion protein-1 (sVAP-1) inflammatory markers (IL-6, TNF-α and high-sensitivity C-reactive protein), which were negatively correlated with epinephrine levels (70). However, the exact underlying mechanism remains to be fully elucidated.

### 6.10 Polycystic ovary syndrome

In patients with PCOS, adropin levels were found to be lower compared to controls, with serum adropin concentrations showing a significant negative correlation with TNF-α levels (71). This suggests that adropin may exert a protective effect against inflammation and the progression of chronic kidney injury in PCOS.

### 6.11 Others

Alterations in adropin levels have also been observed in several immune- and inflammation-related diseases. In multiple sclerosis, a chronic autoimmune disorder, serum adropin levels were markedly reduced (80). Similarly, in patients with rheumatoid arthritis and systemic lupus erythematosus, serum Adropin concentrations were significantly lower than those of healthy controls (72, 73). While the specific mechanisms remain unclear, these findings suggest that adropin could serve as a novel therapeutic target for autoimmune and inflammatory diseases.

## 7 Conclusion and future

Research on the physiological functions of adropin has been ongoing, revealing that adropin levels fluctuate in various physiological and pathological conditions. As a product of the ENHO gene, adropin plays a pivotal role in regulating energy metabolism, particularly in glucose and fatty acid homeostasis (28, 81). Furthermore, adropin has been implicated in cell communication and disease progression by modulating multiple molecular pathways, including NB-3/Notch (20), AKT/CREB/BDNF (37), and VEGFR2/PI3K/AKT (62). In addition, adropin contributes to the pathogenesis of several disorders by influencing immune function, inflammatory responses, and oxidative stress, primarily through the regulation of macrophage metabolism and the modulation of inflammatory cytokine expression. Insufficient adropin levels may lead to immune cell imbalances and elevated pro-inflammatory cytokines, which can impair the immune system’s negative feedback mechanisms, thereby facilitating the initiation of inflammatory processes (Zhang et al., 2020). However, the precise mechanisms underlying these effects remain incompletely understood.

As a relatively recent discovery among regulatory peptides, adropin presents fascinating potential, but both basic and clinical research still face numerous challenges. First, the pharmacokinetics of adropin in circulation remain largely unknown, and the efficacy of peptide hormone administration may be hindered by protein degradation. Furthermore, several aspects of adropin physiology remain unexplored. Second, while adropin expression is influenced by adiposity and various molecules, the specific regulatory mechanisms governing this relationship have yet to be defined. Third, emerging evidence underscores the close association between adropin and various inflammatory diseases, suggesting its involvement in the inflammatory processes of these conditions. Beyond promoting the secretion of inflammatory cytokines, adropin also appears to indirectly regulate the phenotype and biological behavior of immune cells. However, current research mainly focuses on its role in macrophages and Tregs, with insufficient details on the specific mechanisms involved, and little is known about its regulatory effects on other immune cell types. Fourth, clinical studies on adropin have largely been observational, showing correlations between plasma adropin levels and factors such as diet, disease presence, and metabolic parameters (e.g., obesity, coronary heart disease risk, and sex). However, the underlying mechanisms remain unclear. Finally, given that reduced plasma adropin levels are associated with several diseases, including diabetes, atherosclerosis, polycystic ovary syndrome, and multiple sclerosis, and correlate with disease progression, many researchers propose that adropin could serve as a serum biomarker. However, the clinical relevance of any new biomarker must be thoroughly assessed, ensuring that it is suitable for answering key clinical questions. Therefore, large-scale prospective studies involving well-defined populations are essential to establish adropin as a reliable biomarker for various diseases.

In future studies, the pharmacokinetics of adropin in the circulation needs to be further investigated, and the underlying regulatory mechanisms of adropin by molecules such as fat, LXRα, ERα, ROR, and STAT3 need to be further explored. Moreover, research on adropin’s role in regulating inflammation and immunity needs to be increased in order to further explore the mechanism. Clinical research must not only study the mechanism in depth, but also consider whether the therapeutic effect of adropin can be transferred to clinical research. Besides, future research should continue to explore other possible underlying functions of adropin and its analogs, and it is also very meaningful to further study its mechanism of action. Adropin-based treatments may become a new way to treat a variety of diseases.
